# Editorial: Antibody-drug conjugates in solid and hematologic malignancies

**DOI:** 10.3389/fimmu.2025.1662653

**Published:** 2025-08-14

**Authors:** Khalil Saleh, Ayush Raman, Michele Zanoni

**Affiliations:** ^1^ International Department, Gustave Roussy Cancer Campus, Villejuif, France; ^2^ National Cancer Institute, National Institutes of Health (NIH), Bethesda, MD, United States; ^3^ Biosciences Laboratory, Istituto di Ricovero e Cura a Carattere Scientifico (IRCCS) Istituto Romagnolo per lo Studio dei Tumori (IRST) “Dino Amadori”, Meldola, Italy

**Keywords:** antibody-drug conjugates (ADCs), solid tumors, hematologic malignancies, tumor microenvironment, therapy resistance, payload delivery

The therapeutic landscape of hematological and solid tumors has undergone a profound improvement over the last few decades, marked by the advent of immunotherapies such as immune checkpoint inhibitors in solid tumors and lymphomas, as well as adoptive T-cell therapies like chimeric antigen receptor (CAR) T-cells and bispecific antibodies. As part of this revolution, antibody-drug conjugates (ADCs) have rapidly emerged as a novel class of therapeutic agents that combine the specificity of monoclonal antibodies with the cytotoxic potential of chemotherapeutic drugs covalently bonded with an engineered chemical linker (1). This approach allows precise delivery of cytotoxic drugs to cancer cells, taking advantage of the antibody’s specificity for target antigens, thus minimizing systemic toxicity (2). More than 200 ADCs are currently in different stages of clinical trials, with 15 approvals already granted in 2024 in multiple solid tumors, including breast, gynecological, and urothelial cancers, as well as hematologic malignancies (3–7). Despite ADCs holding great promise in cancer treatment, the mechanisms underlying their activity remain still not fully understood. In addition, the clinical efficacy of these agents is often limited by the emergence of acquired resistance and by the poor characterization of their interactions with the tumor microenvironment (TME), constituting a critical barrier to the full realization of their therapeutic potential. This Research Topic includes two reviews and three research articles that examine the mechanisms of action and resistance of several approved or underdeveloped ADCs. Furthermore, they discuss the potential interactions between ADCs and the TME, as well as the predictive factors influencing treatment response. Long et al. have provided an overview of the structural components and functional variations of ADCs, detailing how antibody specificity, linker stability, and payload potency are essential to efficacy. This work also introduces the next-generation ADCs, including bispecific ADCs, probody-drug conjugates, immune-stimulating antibody-drug conjugates (ISACs), degrader-antibody conjugates (DACs), and dual-payload ADCs. These novel designs allow for improved tumor selectivity, payload release, and immunomodulatory activity, thereby addressing limitations inherent to first-generation ADCs. Finally, the authors also emphasize the challenges of off-target effects and systemic toxicities, which remain one of the major issues despite the improved precision of newer ADCs. This critical point is further discussed by the clinical observations of O’Sullivan et al., who reported the development of hepatopulmonary syndrome (HPS) as a novel adverse effect during prolonged treatment with ado-trastuzumab emtansine (T-DM1) in patients with HER2-positive breast cancer. In this case series, four patients who had received long-term treatment with T-DM1 developed hypoxemia and portal hypertension, consistent with HPS, a condition that previously was never associated with ADCs. These findings emphasize the necessity for continuous surveillance and long-term toxicity assessments to chronic exposure to ADCs for the discovery of latent and less common toxicities not revealed during initial clinical trials. Parallel to concerns about safety, ADCs have been shown to be effective in the treatment of tumors refractory to chemotherapy. In a case report, Xue et al. described the rapid and complete remission achieved with gemtuzumab-ozogamicin (GO) in a pediatric patient with refractory systemic mastocytosis and AML::ETO-positive AML. This case highlighted the therapeutic value of GO as an effective salvage therapy in pediatric patients with rare and refractory hematologic malignancies, supporting also further clinical explorations of ADCs in rare and high-risk settings. These data suggest that ADCs are emerging as a promising strategy for the treatment of patients who develop resistance to standard therapies. In bladder cancer, known for its high recurrence and limited treatment response, ADCs have demonstrated remarkable efficacy. In the review article of Zhang and Li, the authors discussed key advances in urothelial carcinoma, with a particular focus on the clinical impact of enfortumab vedotin (targeting NECTIN-4) and sacituzumab govitecan (targeting TROP-2), which have profoundly changed outcomes in patients unresponsive to chemotherapy or immunotherapy. Enfortumab vedotin has dramatically changed the treatment landscape of patients with metastatic urothelial carcinoma in combination with pembrolizumab, a programed-death 1 inhibitor, in a chemotherapy-free regimen that dethroned after several decades of platinum-based chemotherapy in the phase III EV-302 trial in the first-line setting (8). However, based on the negative results of the phase III TROPiCS-04 trial, the FDA and Gilead withdrew approval of sacituzumab govitecan in urothelial carcinoma (9). Furthermore, a potential step forward consists of the integration of ADCs with immune checkpoint inhibitors. Nevertheless, authors report that several challenges still need to be addressed. These include off-target toxicities, antigen heterogeneity, and the need for better patient selection. The future of ADC design and optimization lies in refining their components, enhancing target specificity, and aligning therapies with predictive biomarkers. Despite the expression of the specific target antigen remaining the primary selection criterion for ADC use, its predictive value is often imperfect. Indeed, response to ADC treatment remains heterogeneous, with some patients expressing high levels of the target antigen showing limited benefit while others with lower expression levels experience significant responses. In a case report of Feng et al., a woman with HER2−low metastatic clear−cell endometrial carcinoma, who underwent relapse after surgery, chemotherapy, and radiotherapy, showed a complete response to the HER2-targeting ADC disitamab vedotin. This data suggests that HER2−low endometrial cancers, usually considered ineligible for anti-HER2-targeted therapies, may benefit from ADC approaches. These results underscore the need for more sophisticated biomarker models that might incorporate, for example, antigen density and internalization efficiency. Furthermore, novel approaches of spatial and molecular profiling of tumors, along with liquid biopsy, may offer promising tools to stratify patients and personalize ADC therapy. In conclusion, this Research Topic offers a comprehensive overview of ADCs as a powerful modality in the treatment of both solid and hematologic malignancies that combine the precision of targeted therapies with the potency of cytotoxic drugs. However, significant challenges remain, and these need to be investigated in order to fully unlock the potential of ADCs in cancer therapy.
